# Comparing machine learning and logistic regression methods for predicting hypertension using a combination of gene expression and next-generation sequencing data

**DOI:** 10.1186/s12919-016-0020-2

**Published:** 2016-10-18

**Authors:** Elizabeth Held, Joshua Cape, Nathan Tintle

**Affiliations:** 1 Department of Mathematics, 396 Carver Hall, Iowa State University, Ames, IA 50011 USA; 2 Department of Mathematics and Computer Science, Rhodes College, 2000 N Parkway, Memphis, TN 38112 USA; 3 Department of Mathematics, Statistics and Computer Science, Dordt College, 498 4th Ave NE, Sioux Center, IA 51250 USA

## Abstract

Machine learning methods continue to show promise in the analysis of data from genetic association studies because of the high number of variables relative to the number of observations. However, few best practices exist for the application of these methods. We extend a recently proposed supervised machine learning approach for predicting disease risk by genotypes to be able to incorporate gene expression data and rare variants. We then apply 2 different versions of the approach (radial and linear support vector machines) to simulated data from Genetic Analysis Workshop 19 and compare performance to logistic regression. Method performance was not radically different across the 3 methods, although the linear support vector machine tended to show small gains in predictive ability relative to a radial support vector machine and logistic regression. Importantly, as the number of genes in the models was increased, even when those genes contained causal rare variants, model predictive ability showed a statistically significant decrease in performance for both the radial support vector machine and logistic regression. The linear support vector machine showed more robust performance to the inclusion of additional genes. Further work is needed to evaluate machine learning approaches on larger samples and to evaluate the relative improvement in model prediction from the incorporation of gene expression data.

## Background

Breakthroughs in genome-wide sequencing continue to motivate the development of novel methods to identify risk factors for complex diseases. Machine learning methods (MLMs) are statistical algorithms that allow a computer to learn from one data set (selection set) and make inferences to other data of the same nature. MLMs lend themselves to the genetic analysis of diseases with multiple and complex risk factors, because of the high-dimensional nature of the data. Despite some initial applications of machine learning to genetic association studies with sequence data [1, 2], MLMs remain outside of the mainstream for evaluating genotype–phenotype association.

We extend a recently proposed supervised machine learning approach [3] in order to further understand the behavior and performance of MLMs on sequence data. We incorporated a recent statistical model proposed for the joint analysis of gene expression data and genotype data in evaluating disease risk [4], along with explicit consideration of the analysis of rare variants using a collapsing (burden) approach [5].

## Methods

### Data

Genetic Analysis Workshop 19 (GAW19) provided real DNA sequences and simulated phenotype data on individuals from complex pedigrees. Two-hundred independent simulations of a dichotomous hypertension variable were provided. All simulations were based on the same genetic disease model, and thus have the same set of known, causal variants across a set of fixed genotypes. Our analyses considered different subset of the 200 simulated versions of the variable.

Each simulated version of the hypertension variable is an indicator variable for an individual’s hypertensive status (0 = not hypertensive or 1 = hypertensive). By summing across multiple, independent simulations, we created a more specific disease status variable. In particular, we computed 5 different modified hypertension status variables, *Y*_*i,m*_ as:

$$ Modified\  Hypertension\  Status\left({Y}_{i,m}\right)=\left\{\begin{array}{l}0\  if\frac{l}{m}<0.5\hfill \\ {}1\  if\frac{l}{m}\ge 0.5\hfill \end{array}\right\} $$

where, *l* = number of times individual *i* is classified as hypertensive out of *m* independent simulation replicates. We considered values of *m* = 5, 25, 50, 100, and 150 in our analyses. This resulted in 80, 70, 66, 64, and 65 individuals with *Y*_*i*,*m*_ = 1 out of the total sample of *n =* 637 individuals for *m* = 5, 25, 50, 100, and 150, respectively.

Additionally, we used a series of covariates, including age, sex, pedigree structure, and smoking status. Because evaluating the potential gain in predictive ability when using gene expression data to predict disease risk was a key goal of our study, we limited our analysis to *n* = 637 individuals for whom gene expression data was available. Gene expression data was obtained from peripheral blood mononuclear cells at the first examination period using an Illumina whole-genome expression array.

### Analysis

Following previous work on this data set [3], we use different combinations of the first 150 simulated data sets (SIMPHEN.1 to SIMPHEN.150) to select genes of interest and used 3 other, arbitrarily chosen, simulated data sets (SIMPHEN.197 to SIMPHEN.199) from the remaining simulated sets, as classification data sets. We now provide details of the selection and classification steps.

### Gene selection method

We started by fitting the following model below (1) for each gene in the selection data set. The model is an extension of a recently proposed model [3] but using (a) variant collapsing across the gene and (b) adding main effect and interaction terms for gene expression data.

1 $$ logit\left( \Pr \left(Y=1\right)\right)= Age+ Sex+ Smoke+ Age* Sex+ Pedigree+{G}_i+{S}_i+{G}_i{S}_i $$

where *Y* = 1 indicates that an individual is hypertensive, *Sex* and *Smoke* are indicator variables for the respondent’s Sex and Smoking status, respectively, and *Age* is a continuous measure of the respondent’s age. Pedigree information was incorporated into the model via the use of an indicator variable for each distinct pedigree as has been done previously with this data set [3]. *G*_*i*_ is a continuous measure of gene expression at the gene of interest, *i*, and *S*_*i*_ is an indicator of the presence of any rare (minor allele frequency <5 %) alleles at any location within the same gene of interest, *i*; collapsing is done in the spirit of combined multivariate and collapsing [5]. We also include a gene–single nucleotide polymorphism (SNP) interaction term, *G*_*i*_ *S*_*i*_, as recently proposed [4], to account for potential interactions between expression level and genotype on hypertensive status. Following earlier work exploring the use of support vector machines on this data set [3], the *p* value for each gene containing at least 1 causal variant, as well as randomly selected genes not containing any causal variants, were computed using the model above.

### Classification method

Predictions of disease status were made using 1 of 3 approaches.

### Logistic regression

The first approach, logistic regression (LR), included 1 or more of the most strongly associated causal and/or non-causal genes from the selection step (based on smallest *p* values), and applied eq. (1), with separate terms for each gene, to the classification data set. The result is a LR model which can be used to make predictions of disease status for each individual.

### Support vector machine approaches

The final 2 classification approaches used support vector machines (SVMs) to make classifications. The svm() and tune.svm() functions in R [6] were used to make predictions. In particular, the SVM was provided the prediction model (including 1 or more genes), the selection data set and either a linear or radial basis kernel (the 2 different SVM approaches used). Tenfold cross-validation was used to estimate the kernel hyperparameters, γ and C, and probabilistic estimations of the likelihood of hypertension were allowed.

### Performance evaluation

We considered 315 different combinations of gene lists, phenotype simulations, and gene expression data values. In particular, linear SVM, radial SVM, and LR were applied to lists of the top 1, 5, or 10 causal genes identified at the selection stage, or to the top 5, 15, or 50 non-causal genes, or to models containing no genetic data. To evaluate the impact of variation in gene expression data, which was the same for each person in both the selection and classification data sets, we added random noise (a uniform [−*k, k*] random variable) to observed gene expression values where *k* = 0, 0.01, and 0.1 (3 combinations) in the selection data. Thus, we explored a total of 315 combinations (7 gene lists × 3 levels of gene expression noise × 3 classification data sets × 5 different phenotypes (values of m) = 315). For each combination, all 3 classification models were applied, with area under the receiver operating characteristic curve (AUC) computed for each model-combination based on which individuals were actually hypertensive in the data set.

### Follow-up analyses

Two small-scale follow-up analyses were conducted.

### Follow-up #1

The first follow-up analysis evaluated the value added of gene expression data by fitting models without gene expression data (no gene expression main effect, *G*_*i*_, or interaction term, *G*_*i*_ *S*_*i*_, in the model [eq. 1]) were run on SimPhen.197 with *m* = 5 for 1, 5, and 10 causal genes. All 3 classification methods (LR, radial SVM, and linear SVM) were used.

### Follow-up #2

The second follow-up analysis looked at the impact of the choice of phenotype. Because the collapsed phenotype defined earlier (see Data above) only identifies slightly more than 10 % of subjects as hypertensive (a fairly specific diagnostic measure), we also implemented a more sensitive measure of hypertension. The more sensitive measure of hypertension diagnosis, *Z*_*i,m*_ is defined as:

$$ Sensitive\  Hypertension\  Status\left({Z}_{i,m}\right)=\left\{\begin{array}{l}0\  if\frac{l}{m}=0\hfill \\ {}1\  if\frac{l}{m}>0\hfill \end{array}\right\} $$

where *l* = number of times individual *i* is classified as hypertensive out of *m* independent simulation replicates. We considered values of *m* = 5, 25, 50, 100, and 150 in this follow-up analysis. This follow-up analysis used *k* = 0 for SIMPHEN.197 and considered 1, 5, and 10 causal genes for all 3 classification methods (LR, radial SVM, and linear SVM).

## Results

**Fig. 1:**
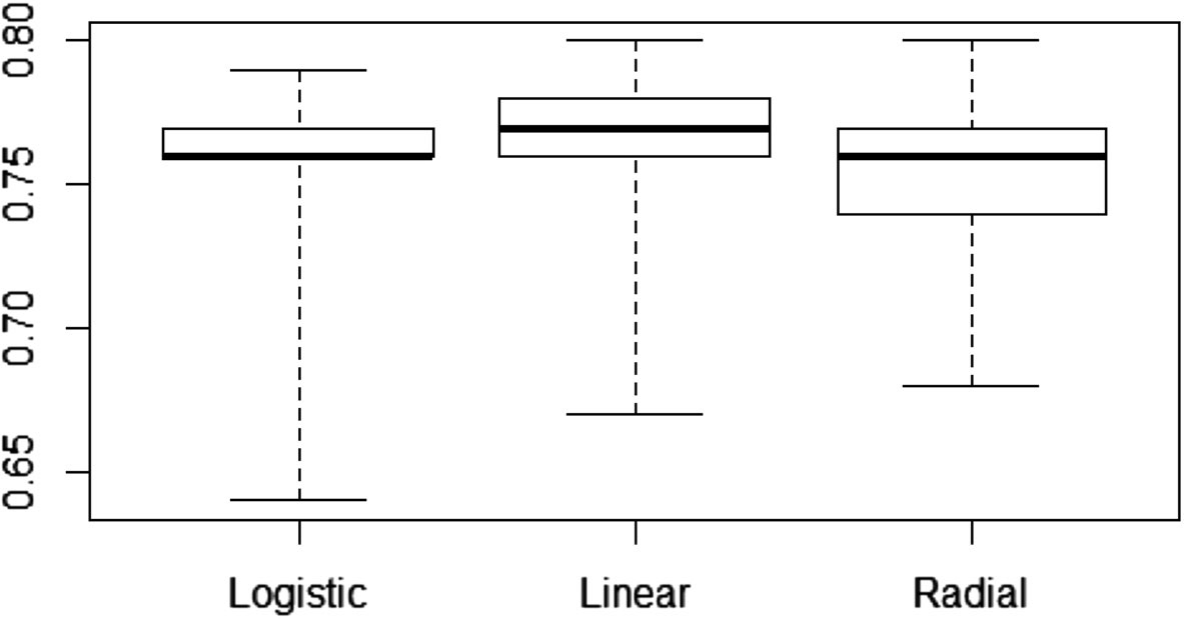
Overall performance (AUC) of the 3 classification approaches across 315 different situations. All 3 methods performed fairly similarly on AUC overall. Linear SVM tended to slightly outperform both other methods across the 315 different settings investigated

Overall, LR, linear SVMs, and radial SVMs yielded similar values of AUC across all 315 settings (Fig. 1) with baseline AUC values (no genetic data, covariates only) of 0.777 (linear), 0.771 (radial), and 0.771 (logistic) on average. Linear SVM tended to outperform both other methods by a slight margin overall (mean improvement vs. LR = 0.009 [SD = 0.016]; mean improvement vs. radial SVM = 0.012 [SD = 0.016]), differences which were statistically significant (*p* <0.001 in both cases). Linear SVM also provided the largest AUC of the 3 methods in the majority of cases examined here (54.9 % = 173/315). Radial SVM (largest AUC in 19.7 % = 62/315 of cases) and LR (largest AUC in 25.4 % = 80/315 of cases) yielded the best AUC in approximately equal numbers in the remainder of cases.

To better understand how different variables affected performance of the 3 different prediction methods, we used a multiple regression model predicting AUC by number of causal genes, number of noncausal genes, *k* (gene expression noise), and *m* (number of phenotypes being collapsed). Separate models were run for each of the 3 different prediction methods. Results summarizing estimated effects of each model parameter and significance are shown in Table 1.

**Table 1:** Regression analysis summarizing association of model parameters and model performance across 315 different situations

	Method		
Model parameters	LR $\widehat{\beta}(SE)$	Radial SVM $\widehat{\beta}(SE)$	Linear SVM $\widehat{\beta}(SE)$
Gene expression noise (*k*)	−3.5 × 10^−2^ (1.2 × 10^−2^)**	−6.4 × 10^−2^ (2.0 × 10^−2^)**	−3.3 × 10^−2^ (1.7 × 10^−2^)
Number of collapsed phenotypes (*m*)	−4 × 10^−5^ (1.1 × 10^−5^)***	−5.49 × 10^−6^ (1.7 × 10^−5^)	−4.4 × 10^−5^ (1.5 × 10^−5^)**
Number of causal genes	−7.5 × 10^−4^ (1.7 × 10^−4^)***	−8.2 × 10^−4^ (2.7 × 10^−4^)**	−1.6 × 10^−4^ (2.3 × 10^−4^)
Number of random genes	−1.7 × 10^−3^ (3.6 × 10^−5^)***	−9.8 × 10^−4^ (5.6 × 10^−5^)***	−1.0 × 10^−3^ (4.8 × 10^−5^)***
Model r^2^	88.4 %	50.7 %	62.5 %

$\widehat{\beta}$, the estimated coefficient in the regression model; SE, the estimated standard error of the coefficient

Regression models predicted AUC by 4 different model parameters for each of the 3 methods separately

Statistical significance of the estimated regression coefficients is indicated by asterisks (***p <0.001, **p <0.01)

In all 3 models, as expected, adding more noncausal genes to the model reduced the AUC. However, the impact of including noncausal genes was approximately twice as much for LR (−1.7 × 10^−3^) as compared to either SVM approach (radial: −9.8 × 10^−4^; linear: −1.0 × 10^−3^). Adding more causal genes to the model also reduced the AUC, although the impact was approximately 5 times less for the linear SVM approach (−1.6 × 10^−4^) and not a statistically significant effect (*p* = 0.47), as compared to radial SVM (−8.2 × 10^−4^) and LR (−7.5 × 10^−4^), where the impact of adding causal genes was similar to the addition of noncausal genes and highly statistically significant (*p* <0.001 in both cases). Finally, all 3 models had reduced predictive ability (lower AUC) in the presence of increased noise in the gene expression data (*k*) and increased numbers of simulations (*m*) collapsed to create the hypertension variable.

### Follow-up analysis #1

Table 2 highlights the impact of including or not including gene expression data for a subset of parameter settings (SIMPHEN.197, *m* = 5). Changes in the AUC were relatively small and variable for all 3 prediction methods.

**Table 2:** Comparing model AUC with and without gene expression data

Number of causal genes	LR		Radial SVM		Linear SVM	
	Without gene exp.	With gene exp.	Without gene exp.	With gene exp.	Without gene exp.	With gene exp.
1	0.775	0.772	0.764	0.764	0.767	0.772
5	0.772	0.773	0.755	0.760	0.759	0.770
10	0.785	0.778	0.739	0.755	0.776	0.770

Model AUC is reported in the table for SIMPHEN.197, *k* = 0 (when expression data was included) and *m* = 5. The table shows that the inclusion of gene expression data had little-to-no impact on the AUC in this data set

### Follow-up analysis #2

Use of a more sensitive phenotype variable (see Methods: Follow-up analyses above) yielded improved AUC values for all 3 prediction methods. Average improvements in AUC were 0.03 (radial: 95 % confidence interval [CI]: 0.01, 0.08), 0.05 (linear: 95 % CI: −0.01, 0.19), and 0.01 (LR: 95 % CI: 0.00, 0.04) across the 15 settings considered in this follow-up analysis.

## Discussion

We have demonstrated a supervised MLM to integrate gene expression data and rare variants in an analysis of disease risk. Linear SVM performed well, likely because of its ability to more robustly handle larger numbers of features (variable) compared to nonlinear SVM and LR. Linear SVM showed the most robustness to the inclusion of both noncausal and causal genes. Notably, AUC decreased for all methods when including additional causal and non-causal genes. This was likely a result of the addition of more variation (noise) than effect (signal) when adding additional genes, regardless of whether or not the genes were causal. Further research is needed to investigate the limits of MLMs with regards to how many genes/features maximizes performance.

While although we sought to potentially increase the predictive ability of the models by evaluating an increasingly specific hypertension variable, the approach resulted in lower predictive ability for all 3 machine learning approaches. Our follow-up analysis showed promise in that increasing the sensitivity of the hypertension variable tended to increase AUC for all 3 methods. This may serve to underscore the fact that optimal statistical study design would have equal numbers of cases and controls when comparing groups on a fixed sample size budget; a particular concern for SVM methods, although some solutions exist [7]. However, whether to use broad (sensitive) or narrow (specific) phenotype definitions remains an important and open question in statistical genetics.

Finally, the inclusion of gene expression data with genotype data at the same loci, generally reduced predictive ability. However, in a follow-up analysis when we compared models with and without gene expression data, but both containing genotype data, results were inconclusive with regards to model predictive ability improvements because of the addition of gene expression data. More work is needed to develop models incorporating gene expression data that directly connect biological mechanisms with the statistical model. We note that in this data set, it is unclear whether the gene expression data actually would be beneficial in large amounts given the simulated nature of the data. Additional exploration is needed with different simulated data sets to quantify the size of effects needed in expression data to be detected by MLMs.

Some additional limitations of our analysis are worth noting. First, although we followed previous researchers in how we incorporated pedigree status in the model [3], more sophisticated approaches may yield better performance (eg, extreme phenotype sampling). Second, the sample size we used was quite small and so the power is likely quite limited for any approaches and consideration of the performance of these methods on larger samples should be considered by future researchers in this area, to see if larger improvements in AUC can be realized. However, despite the decreasing cost of sequencing data, small sample sizes will likely to continue to be a limiting factor in genetic analysis for the short term. Third, we only considered intragenic SNPs, even though intergenic SNPs may be associated with gene expression levels and should be considered in follow-up studies. Finally, the approach we used to compare methods used different simulated data sets provided by the Genetic Analysis Workshop organizers; these data sets, however, have fixed genotypes. Additional simulation studies using with variable genotypes are needed.

## Conclusions

Supervised MLMs continue to be an enticing alternative to mainstream statistical techniques for elucidating genotype-phenotype relationships in large, complex data sets. Further work is needed to develop best practices for such approaches and quantify performance gains vs. standard approaches.
